# Memorial to the Late Dr. Norman Lloyd Price

**Published:** 1954-10

**Authors:** Beryl D. Corner, Arthur L. Eyre-Brook, Francis J. W. Lewis


### *Correspondence*

### Memorial to the late Dr. Norman Lloyd Price

Dr. Norman Lloyd Price was Honorary Physician to Out Patients at the Bristol Royal Hospital for Sick Children from 1937 and the first Shaw Lecturer in Diseases of Children at Bristol University until his death in 1945. He was keenly interested in the development of paediatric services in Bristol, and had looked forward with great enthusiasm to the establishment of the University Department of Child Health and during the short time available to him, he made a very substantial contribution to the practice and teaching of paediatrics in the City.

About six years ago we opened a fund to provide a memorial to him and it seemed appropriate that this should be devoted to some purpose which would further the development of his chosen branch of medicine. We originally suggested that the memorial should take the form of a prize in Child Health for undergraduates at Bristol University. However, since subscriptions were originally invited, the University has received other endowments for this purpose. After discussion it was decided, therefore, that the most fitting memorial would be an endowment for the purchase of books for the section of Child Health and Paediatrics in the University Medical Library. This would benefit a large number of students and practitioners in Bristol and the South West.

Many friends, relatives and members of the Medical and Nursing professions have sent contributions so that recently we were able to give to the University the sum of £300. This has been very gratefully accepted by the Council and the income from this endowment will be used for the purchase of books for the medical library; each will bear an inscription stating that it has been purchased from the Norman Lloyd Price Memorial Fund.

Beryl D. Corner.

Arthur L. Eyre-Brook.

Francis J. W. Lewis.

